# Hot-Deformation Behavior of High-Nitrogen Austenitic Stainless Steel under Continuous Cooling: Physical Simulation of Surface Microstructure Evolution of Superheavy Forgings during Hot Forging

**DOI:** 10.3390/ma12071175

**Published:** 2019-04-10

**Authors:** Zhenhua Wang, Yong Wang

**Affiliations:** 1 School of Mechanical Engineering, Yanshan University, Qinhuangdao 066004, China; 528812958@stumail.ysu.edu.cn; 2 State Key Laboratory of Metastable Materials Science and Technology, Yanshan University, Qinhuangdao 066004, China

**Keywords:** high-nitrogen austenitic stainless steel, superheavy forging, continuous cooling, microstructure evolution, dislocation

## Abstract

Superheavy forgings are increasingly used in the nuclear industry. The strain rate is extremely low during hot forging due to the huge size of the superheavy forging; in fact, the surface temperature of the forging decreases obviously during each deformation step. Hot-deformation behavior differs from that of isothermal deformation. In this study, 18Mn18Cr0.6N steel was selected as a model material. Hot-compression tests were conducted using a Gleeble 3800 simulator at a strain rate of 10^−4^ s^−1^ and continuous cooling rates of 0.0125 Ks^−1^ and 0.025 Ks^−1^. The microstructure was observed using electron backscatter diffraction analysis and transmission electron microscopy. The flow stress increased with increasing strain: the higher the cooling rate, the higher was the hardening rate. Continuous cooling inhibited dynamic recrystallization by delaying its nucleation. The subgrain/cell size increased linearly with increasing final temperature of deformation in the temperature range 1273 to 1448 K. An intense <001> texture formed in 0.8-strained specimens and the matrix exhibited a low Taylor factor orientation. Most dislocations were separately distributed in subgrains and did not entangle with each other or with the subgrain boundary. Dislocation arrays transferred easily through boundaries and dislocation accumulation at boundaries was weak. This study contributes to understanding the hot-forging process of superheavy forgings.

## 1. Introduction

Temperature affects the deformation, dynamic recovery (DRV), and dynamic recrystallization (DRX) mechanisms of metals during hot deformation, as reviewed by Sakai et al. [1]. For most metal products, hot-forming processes, such as rolling, extrusion, drawing, and mechanical press forging, are conducted at a high strain rate, usually above 1 s^−1^, as shown by Dieter et al. [2]. Because of the high strain rate, each deformation step can be considered as an isothermal forming process. The heat loss caused by conduction through dies, rollers, tools, and other components, radiation, and convection is negligible. Therefore, current research mainly concerns the hot-deformation behavior of metals at constant temperatures, as reported by Doherty et al. [3]. Systematic descriptions on the hot-deformation behavior, such as the hot-deformation equation [4], hot-processing maps [5], Zener–Hollomon parameter [6], and DRX grain size model [7] are established under isothermal conditions. 

For common heavy forgings, the forging process is usually conducted using a hydraulic press. Wang et al. [8] indicated that the strain rate is low, typically at 10^−3^ s^−1^. However, the production of superheavy forgings that are used in the nuclear industry, such as nozzle shells, the upper head of reactor pressure vessels, and monoblock low-pressure rotors, needs ingots heavier than 600 t [9,10]. Wang et al. stated [11] that the strain rate during upsetting is far below that generally used in common heavy hot forging, and can be lowered to 10^−4^ s^−1^. Under such a low strain rate, the surface temperature of the forging decreases obviously during each deformation step. To improve the efficiency, safety, and service life of nuclear power plants, superheavy forgings are increasingly used to replace welded structures [12,13,14]. Many characteristics of superheavy forgings, such as their fatigue properties and corrosion resistance, are affected by their surface microstructure. The evolution of surface microstructure of superheavy forgings is, however, still unclear due to their continuous cooling profile and extremely low strain rate. In addition, the finite element method of the industrial forging simulation is the powerful tool for the metallurgical technology development, as presented by Ma et al. [15]. The modeling of hot deformation by finite element method requires the material parameters obtained through physical simulation. 

In this study, a typical high-nitrogen austenitic stainless steel, 18Mn18Cr0.6N, was selected as a model material. This steel contains N and Mn instead of Ni. N is an effective solution strengthener, an austenite stabilizer, and a corrosion-resistance enhancer, as reviewed by Simmons [16]. The flow behavior and microstructure evolution of 18Mn18Cr0.6N steel during hot compression under continuous cooling were investigated. The aim of this study was to improve understanding of the surface microstructure evolution of superheavy forgings during hot forging.

## 2. Materials and Methods 

The 18Mn18Cr0.6N steel was prepared by induction melting and electroslag remelting. The chemical composition was (wt.%): 0.084 C, 17.9 Mn, 18.06 Cr, 0.62 N, 0.46 Si, 0.2 Ni, 0.009 P, and 0.002 S, and the balance was Fe. After solution heat treatment at 1473 K for 5 h, a small slab was cut from the ingot and then hot-rolled at 1371 K to induce a fully recrystallized microstructure. The hot-rolled slab was further heat-treated at 1473 K for 3 h. A single austenite microstructure with an average grain size of 305 μm was obtained. 

Hot-compression testing was conducted using a Gleeble 3800 simulator (Dynamic Systems Inc., Poestenkill, NY, USA). The end of the compression specimen (Φ 10 mm × 18 mm) was lubricated by a tantalum slice and MoS_2_. Hot compression was carried out at 10^−4^ s^−1^ under continuous cooling conditions. The initial deformation temperature was 1473 K and the cooling rates were 0.0125 Ks^−1^ and 0.025 Ks^−1^. Under each cooling condition, specimens were compressed to strains of 0.2, 0.4, and 0.8. After compression, the specimens were water-cooled to freeze the microstructure. 

Each water-cooled specimen was cut along the compression direction. After grinding using 4000 grit SiC paper, the sectioned specimen was polished for 3 h using a chemo-mechanical slurry containing colloidal silica (20 nm). The microstructure was observed using electron backscatter diffraction (EBSD) analysis with TSL-OIM-Analysis software (version 7, EDAX, Mahwah, NJ, USA). Grain orientation spread (GOS) was determined by calculating the average deviation between the orientation of each point in a grain and the average orientation for the grain. A grain was defined as a region surrounded by the boundaries which have misorientation larger than the default value of 5°. Taylor factor maps were calculated. The uniaxial load of specimen was parallel to Axis 1 in the microscope. Therefore, a 90° rotation of the data about Axis 2 was performed after scanning to get it into the right reference frame according to OIM Analysis Help [17].

The dislocation and subgrain structures were studied by transmission electron microscopy (TEM) using a JEM-2010 instrument (JEOL Co., Ltd., Tokyo, Japan). Thin foils were cut from the specimens along the compression direction. The final thinning was achieved by electro-polishing using a bath of acetic acid and 10% perchloric acid.

## 3. Results and Discussion

### 3.1. Flow Behavior

**Figure 1:**
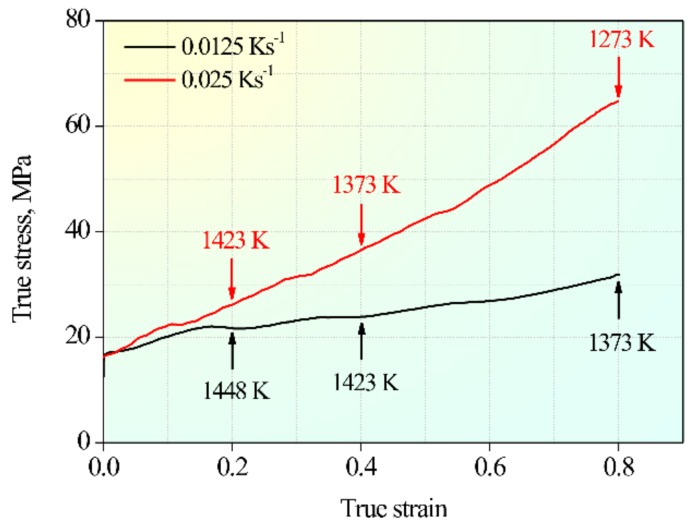
Flow curves of 18Mn18Cr0.6N steel compressed at 10^−4^ s^−1^ under different cooling rate conditions.

Figure 1 shows the flow curves of 18Mn18Cr0.6N steel compressed at 10^−4^ s^−1^ under different cooling rate conditions. The black curve was obtained at a cooling rate of 0.0125 Ks^−1^ and the red curve at 0.025 Ks^−1^. The instantaneous temperatures at different strains are marked on the curves. Under both cooling rate conditions, the flow stress increased with increasing strain. The flow curves were of the work-hardening type, rather than DRX or DRV type. The higher the cooling rate, the higher was the hardening rate.

### 3.2. Microstructure Evolution

**Figure 2:**
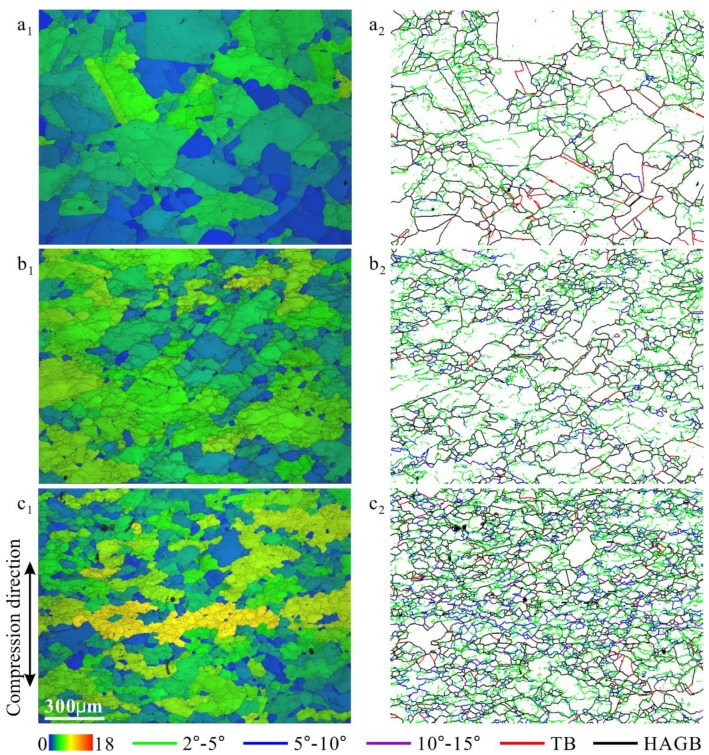
Microstructures of 18Mn18Cr0.6N steel compressed at 10^−4^ s^−1^ and a cooling rate 0.0125 Ks^−1^ to different strains: 0.2 (**a_1_**,**a_2_**), 0.4 (**b_1_**,**b_2_**), and 0.8 (**c_1_**,**c_2_**). (**a_1_**), (**b_1_**), and (**c_1_**) are band contrast images overlaid by grain orientation spread images; (**a_2_**), (**b_2_**), and (**c_2_**) are grain boundary characteristic distribution images. The vertical direction is the compression direction.

Figure 2 shows the microstructure of 18Mn18Cr0.6N steel compressed at 10^−4^ s^−1^ and a cooling rate 0.0125 Ks^−1^ to different strains. a_1_, b_1_, and c_1_ are band contrast images overlaid by GOS images; a_2_, b_2_, and c_2_ are grain boundary characteristic distribution (GBCD) images. In the GOS images, the grains are shaded using different colors. The basic colors of blue, green, yellow, and red represent strains from lowest to highest. In the GBCD images, high-angle grain boundaries (HAGBs) (misorientations ≥ 15°) are shown as black lines; twin boundaries (Σ3) are shown as red lines; low-angle grain boundaries (LAGBs) are shown as green lines (2° ≤ misorientations < 5°), blue lines (5 ≤ misorientations < 10°), and purple lines (10° ≤ misorientations < 15°). 

In the 0.2-strained specimen (Figure 2a_1_), subgrains/cells appeared in the deformed grains. In the corresponding GBCD image (Figure 2a_2_), most LAGBs were green (2° ≤ misorientations < 5°). A few blue LAGBs (5 ≤ misorientations < 10°) can be seen near triple junctions. Several DRX grains formed containing annealing twins. When the strain increased to 0.4 (Figure 2b_1_), most grains were deformed. In the corresponding GBCD image (Figure 2b_2_), the numbers of blue (5 ≤ misorientations < 10°) and purple (10° ≤ misorientations < 15°) LAGBs and DRX grains were all larger than those in the 0.2-strained specimen. Only the number of twin boundaries decreased with increasing strain. When the strain increased to 0.8 (Figure 2c_1_), highly strained grains (yellow) were seen. The subgrain/cell sizes were smaller than those in Figure 2(a_1_,b_1_). 

**Figure 3:**
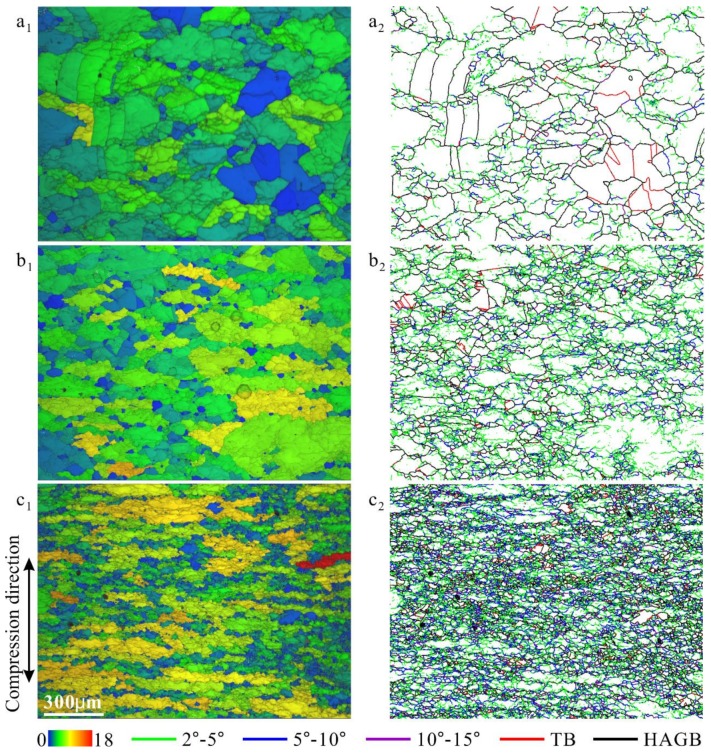
Microstructures of 18Mn18Cr0.6N steel compressed at 10^−4^ s^−1^ and a cooling rate 0.025 Ks^−1^ to different strains: 0.2 (**a_1_**,**a_2_**), 0.4 (**b_1_**,**b_2_**), and 0.8 (**c_1_**,**c_2_**). (**a_1_**), (**b_1_**), and (**c_1_**) are band contrast images overlaid by grain orientation spread images; (**a_2_**), (**b_2_**), and (**c_2_**) are grain boundary characteristic distribution images. The vertical direction is the compression direction.

Figure 3 shows the microstructure of 18Mn18Cr0.6N steel compressed at 10^−4^ s^−1^ and a cooling rate 0.025 Ks^−1^ to different strains. The microstructure evolution trend with increasing strain is similar to that shown in Figure 2; however, due to the higher cooling rate, the strain levels in most grains were higher under the same strain condition. In addition, the subgrain/cell and DRX grain sizes are both finer in Figure 3 than those in Figure 2.

**Figure 4:**
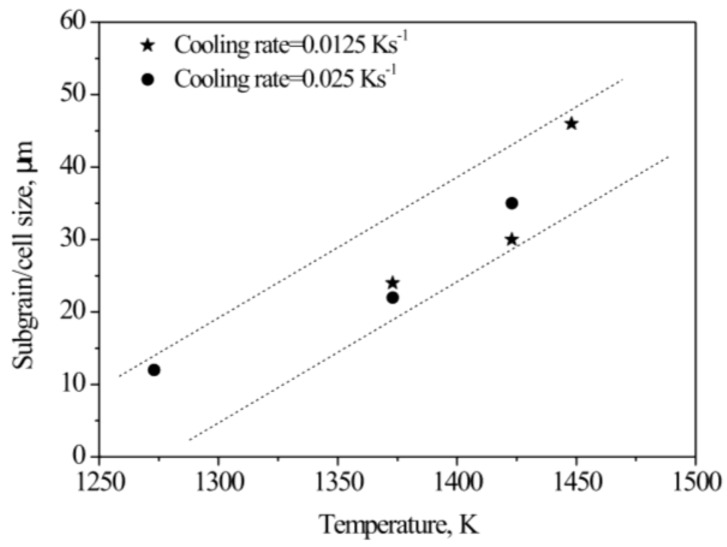
Relationship between subgrain/cell size and final temperature of deformation.

Figure 4 shows the relationship between subgrain/cell size and the final temperature of deformation. The points represent measured data and the dashed lines show the corresponding variation trend. Obviously, the higher the final temperature of deformation, the larger was the subgrain/cell size: this relationship was essentially linear in the temperature range 1273 to 1448 K.

**Figure 5:**
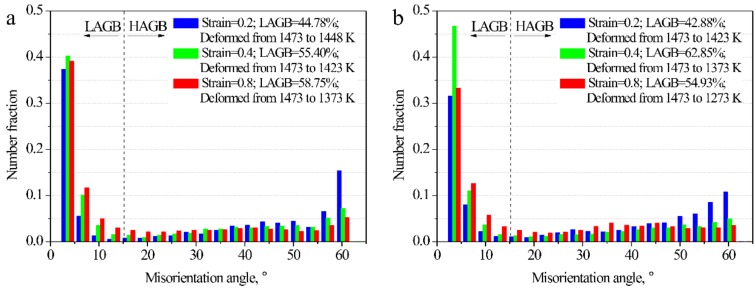
Distribution of misorientation angles of specimens deformed at 10^−4^ s^−1^ with cooling rates of (**a**) 0.0125 Ks^−1^ and (**b**) 0.025 Ks^−1^.

Figure 5 shows the distribution of misorientation angles of specimens deformed at different cooling rates. At a cooling rate of 0.0125 Ks^−1^ (Figure 5a), the higher the strain, the larger was the number fraction of LAGB (increasing from 44.78% to 58.75% for strains of 0.2 to 0.8). At a cooling rate of 0.025 Ks^−1^ (Figure 5b), the highest number fraction of LAGB was in the 0.4-strained specimen (62.85%). Under both cooling rate conditions, the fraction of HAGB was largest in the 0.2-strained specimen. The density of twins in the 0.2-strained specimen was high because the strain was not considerably high to destroy the existing twins in the parent grains. There was inadequate DRX in the 0.8-strained specimen.

**Figure 6:**
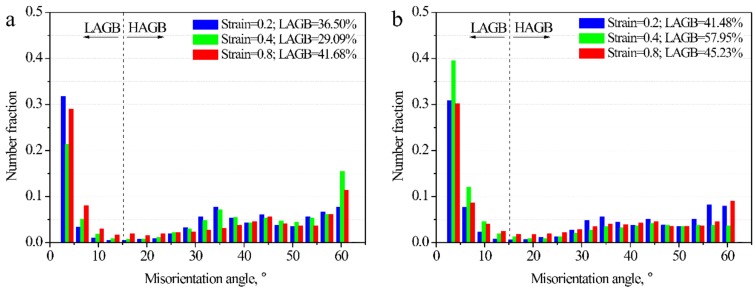
Distribution of misorientation angles of specimens deformed at 10^−^^3^ s^−1^ and different constant temperatures: (**a**) 1423 K; (**b**) 1323 K.

To analyze the effect of continuous cooling on DRX, the experimental data obtained by isothermal compression tests in our previous study [11] were further examined, as shown in Figure 6. At 1423 K, the number fraction of LAGB was lowest in the 0.4-strained specimen (Figure 6a). The densities of twins in the 0.4- and 0.8-strained specimens were high because a lot of DRX grains appeared and twins were formed during the growth of them [11]. At 1323 K, the number fractions of LAGB were similar in the 0.2-strained and 0.8-strianed specimens (Figure 6b). From the data in Figure 5 and Figure 6, it was clearly found that at the same strain rate and strain, and at a similar deformation temperature, the number fraction of LAGB was higher in the continuously cooled specimens: in other words, continuous cooling suppressed DRX. This phenomenon is consistent with earlier observation [8] that continuous cooling (0.4 Ks^−1^) suppressed DRX and deteriorated hot ductility at a strain rate of 10^−3^ s^−1^.

### 3.3. Texture and Taylor Factor

**Figure 7:**
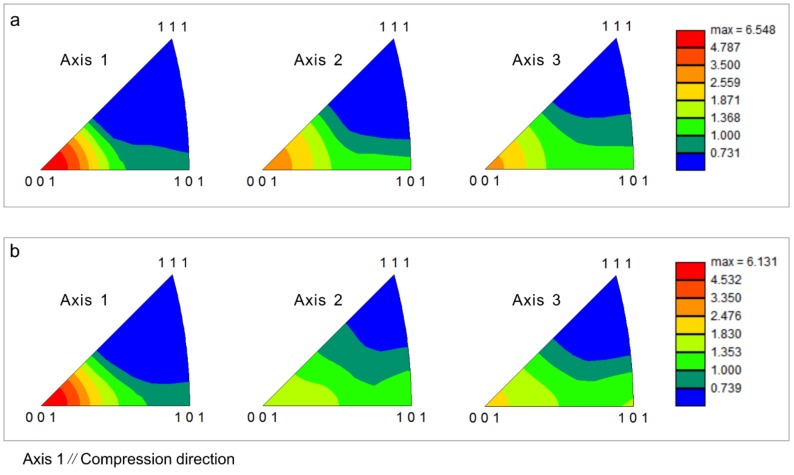
Inverse pole figures of 18Mn18Cr0.6N steel compressed to a strain of 0.8 at different cooling rates: (**a**) 0.0125 Ks^−1^; (**b**) 0.025 Ks^−1^.

Texture usually forms in deformed specimens where DRX is not adequate. The inverse pole figures of 18Mn18Cr0.6N steel compressed to a strain of 0.8 under continuous cooling are shown in Figure 7. Axis 1 was parallel the compression direction during EBSD examination. An intense <001> fiber texture formed in both 0.8-strained specimens. The maximum intensities were above 6 under both cooling rate conditions.

**Figure 8:**
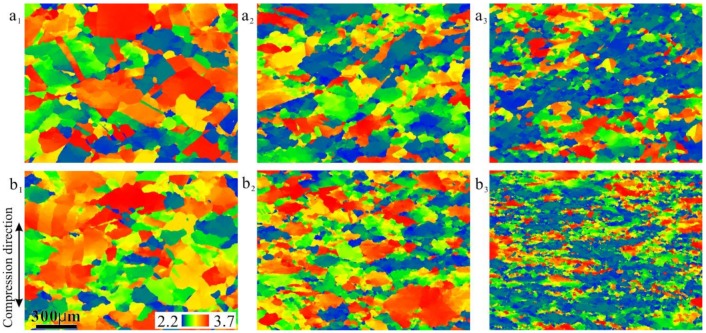
Taylor factors of 18Mn18Cr0.6N steel compressed at different cooling rates: (**a_1_**–**a_3_**) 0.0125 Ks^−1^; (**b_1_**–**b_3_**) 0.025 Ks^−1^. (**a_1_**) and (**b_1_**) are at a strain of 0.2; (**a_2_**) and (**b_2_**) are at a strain of 0.4; (**a_3_**) and (**b_3_**) are at a strain of 0.8. The vertical direction is the compression direction.

**Figure 9:**
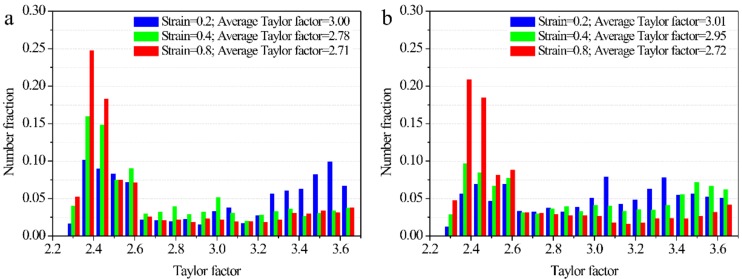
Distribution of Taylor factors of specimens deformed at cooling rates of (**a**) 0.0125 Ks^−1^ and (**b**) 0.025 Ks^−1.^

Figure 8 shows the Taylor factor images of specimens deformed under continuous cooling. The Taylor factor were used in the analysis of the plastic deformation of polycrystalline metals and implies the distribution of the grain orientations; grains could be classified into “hard” and “soft” based on their Taylor factors [17,18]. At strains of 0.2 and 0.4 under both cooling rates, the distributions of Taylor factors were random (Figure 8(a_1_,a_2_,b_1_,b_2_)); however, there were large areas with low Taylor factors (blue and green colors) in the 0.8-strained specimens (Figure 8(a_3_,b_3_)). The smaller the Taylor factor, the ‘softer’ was the grain. Figure 9 shows quantitative results for the Taylor factor distributions. Under both cooling rate conditions, the higher the strain, the smaller was the average Taylor factor. Although the DRX content was small, the extremely low strain rate gave the matrix enough time to recovery and then the matrix evolved to a soft orientation to coordinate deformation through DRV and rotation. It should be noted that the statement of a soft orientation was based on the Taylor factor data. Detailed micro-texture analysis is needed to support it in further study. 

### 3.4. TEM Microstructures

**Figure 10:**
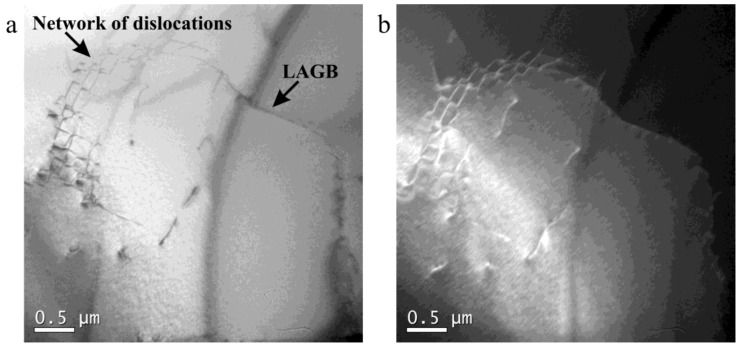
Transmission electron microscopy microstructure of 18Mn18Cr0.6N steel compressed to a strain of 0.4 at a cooling rate of 0.0125 Ks^−1^: (**a**) bright-field image; (**b**) corresponding dark-field image.

Figure 10 shows the TEM microstructure of 18Mn18Cr0.6N steel compressed to a strain of 0.4 at a cooling rate of 0.0125 Ks^−1^. The dislocations entangled and formed a network of dislocations (Figure 10a). The network of dislocations connected with a LAGB, which was obviously constructed by or evolved from dislocations. The subgrain size exceeded 5 μm. Figure 10b shows the corresponding dark-field image. Based on the data presented in Figure 2, Figure 3, and Figure 10, the DRV process can be known: Dislocations form in the early stage of deformation. As deformation develops, dislocations become entangled, and cell structures and subgrains are formed. In the late deformation stage, subgrains will rotate, and their misorientation angle will increase.

**Figure 11:**
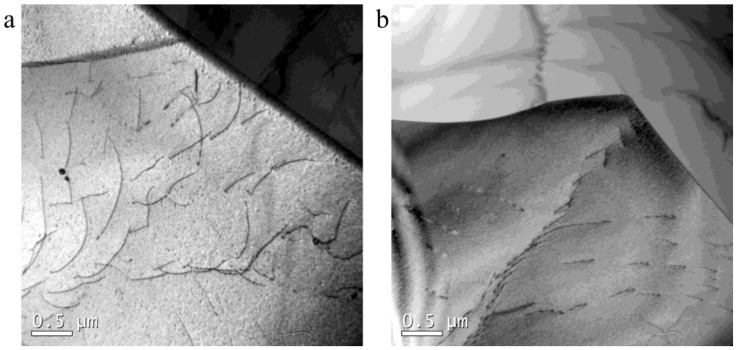
Dislocation morphology in subgrain in 18Mn18Cr0.6N steel compressed at a cooling rate of 0.0125 Ks^−1^ to different strains: (**a**) 0.4; (**b**) 0.8.

**Figure 12:**
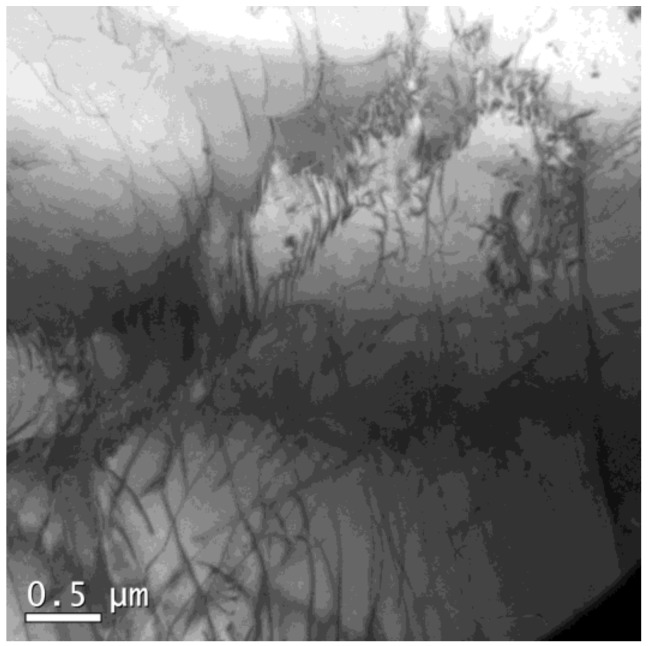
Dislocation morphology in subgrain in 18Mn18Cr0.6N steel compressed at a cooling rate of 0.025 Ks^−1^ to a strain of 0.8.

Wang et al. reported [8] that subgrains formed at high temperature are larger and have no energy advantage to transform to DRX nuclei in comparison with those formed at low temperature. It was therefore of interest to understand the evolution of subgrains formed in the early stage of compression (at high temperature) during the subsequent (low-temperature) deformation stage. Figure 11 shows the dislocation morphology in a subgrain in 18Mn18Cr0.6N steel compressed at a cooling rate of 0.0125 Ks^−1^ to different strains. Dislocations distributed separately in the subgrain. Most dislocations were single and did not entangle with each other or with the subgrain boundary. Similar dislocation morphology was found in a subgrain in 18Mn18Cr0.6N steel compressed at a cooling rate of 0.025 Ks^−1^ to a strain of 0.8 (Figure 12). The dislocation density was very high in the subgrain. This is only a qualitative assumption which is based on simple observation without considering any known crystallographic approaches. These separately distributed dislocations provided no obvious beneficial effect to subgrain rotation or DRX nuclei formation. 

**Figure 13:**
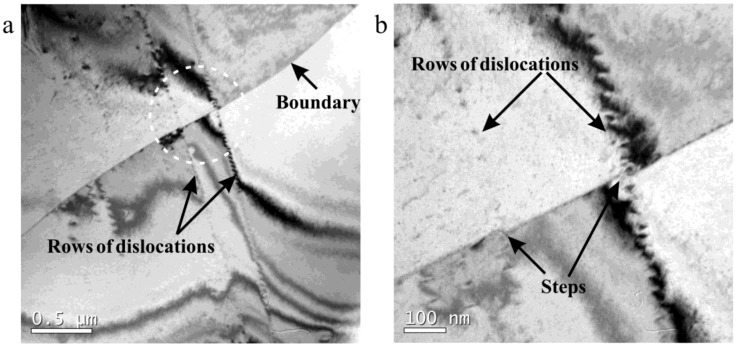
Dislocation morphology in 18Mn18Cr0.6N compressed at a cooling rate of 0.025 Ks^−1^ to a strain of 0.4: (**b**) is a higher magnification of the marked circle region in (**a**).

**Figure 14:**
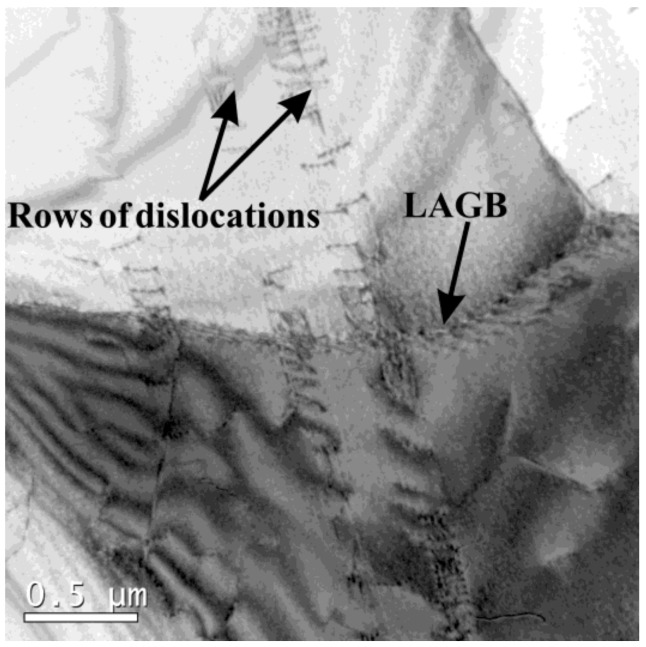
Dislocation morphology in 18Mn18Cr0.6N compressed at a cooling rate of 0.025 Ks^−1^ to a strain of 0.4.

In addition, dislocation arrays were found in a deformed specimen, as shown in Figure 13 and Figure 14. In Figure 13a, dislocation arrays transferred through a boundary (HAGB or twin boundary), similar to that of a cold-deformed microstructure in 18Mn18Cr0.5N steel investigated by Jandová et al. [19]. Figure 13b shows that the dislocation arrays induced steps on the boundary. Figure 14 shows the transfer of dislocation arrays through an LAGB. Dislocation accumulation at boundaries was obviously weak in this study, which made it difficult for DRX nucleation to occur. 

**Figure 15:**
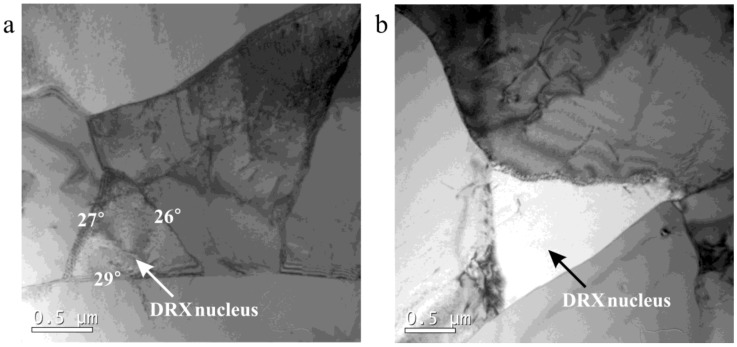
Dynamic recrystallization nuclei at triple junctions in 18Mn18Cr0.6N compressed to a strain of 0.8 at different cooling rates: (**a**) 0.0125 Ks^−1^; (**b**) 0.025 Ks^−1.^

Miura et al. [20] found that DRX occurs easily at triple junctions. In this study, DRX nuclei were found at triple junctions, as shown in Figure 15. These two DRX nuclei were triple shaped and had straight boundaries, which meant that their migration rate would be low. The sizes of these two DRX nuclei at triple junctions were much smaller than that of the subgrains. This can be explained by the theory proposed by Wang et al. [21], in which it was reported that DRX grain sizes formed at triple junctions are finer because these have higher energy than other locations.

Huang and Logé [22] indicated that many factors affect DRX behavior. To thoroughly understand the effect of continuous cooling on DRX, the comprehensive influences of initial grain size, stacking fault energy, and strain rate should be carefully considered in further work. 

## 4. Conclusions

(1) Flow stress increased with increasing strain: the higher the cooling rate, the higher was the hardening rate.
(2) Continuous cooling inhibited DRX by delaying its nucleation.
(3) Subgrain/cell size increased linearly with increasing final temperature of deformation in the temperature range 1273 to 1448 K.
(4) An intense <001> texture was formed in 0.8-strained specimens and evaluation of the matrix showed a low Taylor factor orientation.
(5) Most dislocations were separately distributed in subgrains and did not entangle with each other or with the subgrain boundary. Dislocation arrays transferred easily through boundaries and dislocation accumulation at boundaries was weak.
(6) DRX nuclei at triple junctions were much smaller than normal subgrains.
